# Deucravacitinib for refractory lichen planus with post-inflammatory hyperpigmentation in skin of color

**DOI:** 10.1016/j.jdcr.2026.08.006

**Published:** 2026-08-17

**Authors:** Aditya Dutt, Mallory Suhling, Aliya Mirza, Christopher Logas

**Affiliations:** a Chicago College of Osteopathic Medicine, Midwestern University, Downers Grove, Illinois; b Innovative Dermatology and Mohs Surgery, Bonita Springs, Florida; c Illinois Dermatology Institute, Skokie, Illinois

**Keywords:** biologic, deucravacitinib, hyperpigmentation, jak, janus kinase inhibitor, lichen planus, skin of color, TYK2 inhibitor

## Introduction

Lichen planus (LP) is an inflammatory T-cell-mediated disease affecting the stratified squamous epithelium. Characteristic features include overlying white, reticulated lines termed Wickham’s striae; the classic “6 Ps”—purple, polygonal, planar, pruritic, papular, and plaque-like lesions; and the development of post-inflammatory hyperpigmentation (PIH) in recurrent cases.^1^ LP refractory to corticosteroid treatment has led dermatologists to prescribe off-label treatments targeting immune pathways, such as calcineurin, interleukin-12 (IL-12), interleukin-17 (IL-17), interleukin-23 (IL-23), tumor necrosis factor alpha (TNF-α), and Janus kinase (JAK) inhibitors.^1^ More recently, deucravacitinib (SOTYKTU; Bristol-Myers Squibb, Princeton, NJ, USA), a selective tyrosine kinase 2 (TYK2) inhibitor FDA approved for plaque psoriasis, has been used successfully off-label in the treatment of diffuse, hypertrophic, oral, and vulvar LP.^2^, ^3^, ^4^, ^5^ There is a paucity of cases in the literature detailing the usage of deucravacitinib for recurrent LP and consequent PIH, especially in skin of color (SoC). We report a case of chronic, diffuse LP with PIH and significant pruritus controlled with deucravacitinib.

## Case report

A 78-year-old South Asian woman with a past medical history of hay fever presented to the dermatology clinic with worsening hyperpigmented, pruritic plaques distributed on her trunk and bilateral extremities (Fig 1, *A* and *C*). The lesions involved approximately 15% of her body surface area (BSA), with pruritus rated 4/10 on a numeric rating scale. Punch biopsies of the right wrist and left thigh were performed. She was started on triamcinolone acetonide 0.1% cream twice daily for 2 weeks for suspected LP.

**Fig 1:**
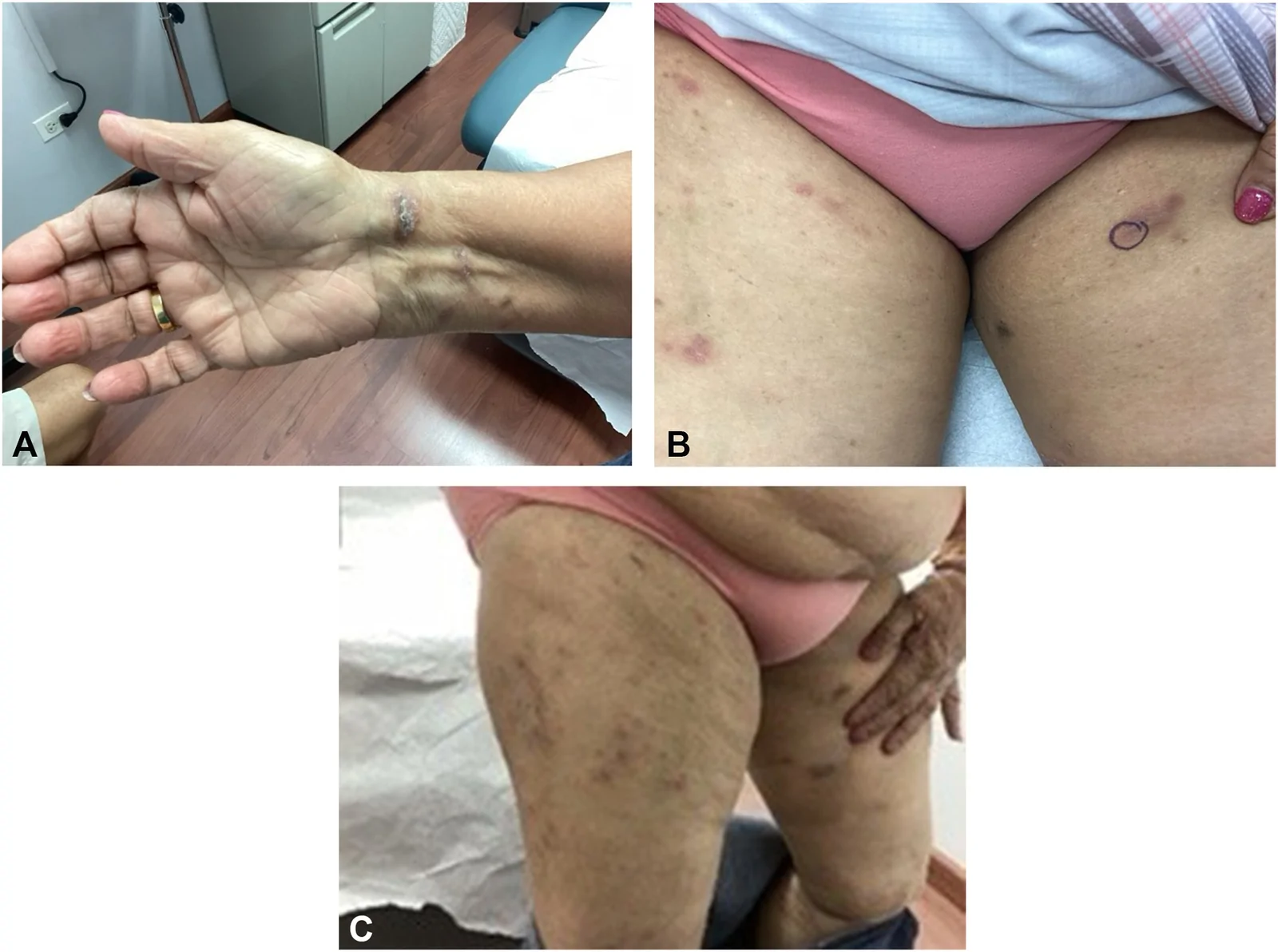
Diffuse hyperpigmented lesions at initial presentation. **A,** Hyperpigmented, scaly plaque on the right ventrolateral wrist. **B,** Hyperpigmented patch on the left inguinal thigh (biopsy taken from the circled area). **C,** Diffuse hyperpigmented plaques and patches on the bilateral thighs.

2 weeks later, biopsy results confirmed diffuse LP. A hepatitis C panel was negative. Treatment was escalated to include clobetasol 0.05% cream twice daily (2 weeks on with 1-week breaks), tretinoin 0.05% cream daily, and metronidazole 500 mg orally once daily for 1 week, followed by twice daily thereafter. Triamcinolone was continued, and phototherapy was recommended; however, the patient was unable to commit to the treatment schedule required to achieve therapeutic benefit with narrowband UVB.

After 2 months, the patient’s lesions and symptoms remained unresponsive to the aggressive topical and systemic regimen. The patient voiced concern regarding lesion progression and worsening nocturnal itch. She declined oral corticosteroids due to previous adverse effects, including insomnia and mood changes, experienced during prior treatment for unrelated conditions. Methotrexate was not pursued because the patient declined complete alcohol cessation. Additionally, oral isotretinoin was discussed as an alternative therapy, but the patient preferred a treatment with more rapid relief of pruritus.

All previous therapies were discontinued. She was transitioned to deucravacitinib 6 mg orally once daily. Baseline laboratory tests obtained prior to initiation included a complete blood count with differential, lipid panel, and interferon-gamma release assay. At her 1-month follow-up, she reported significant pruritus relief (1/10), and her lesions decreased to 9% BSA. Tretinoin 0.05% cream daily was resumed to address persistent hyperpigmentation. At 12-week follow-up, the patient demonstrated near-complete clinical resolution. Her lesions were reduced to 2% BSA with no associated pruritus (0/10) and markedly improved hyperpigmentation (Fig 2, *A* and *C*).

**Fig 2:**
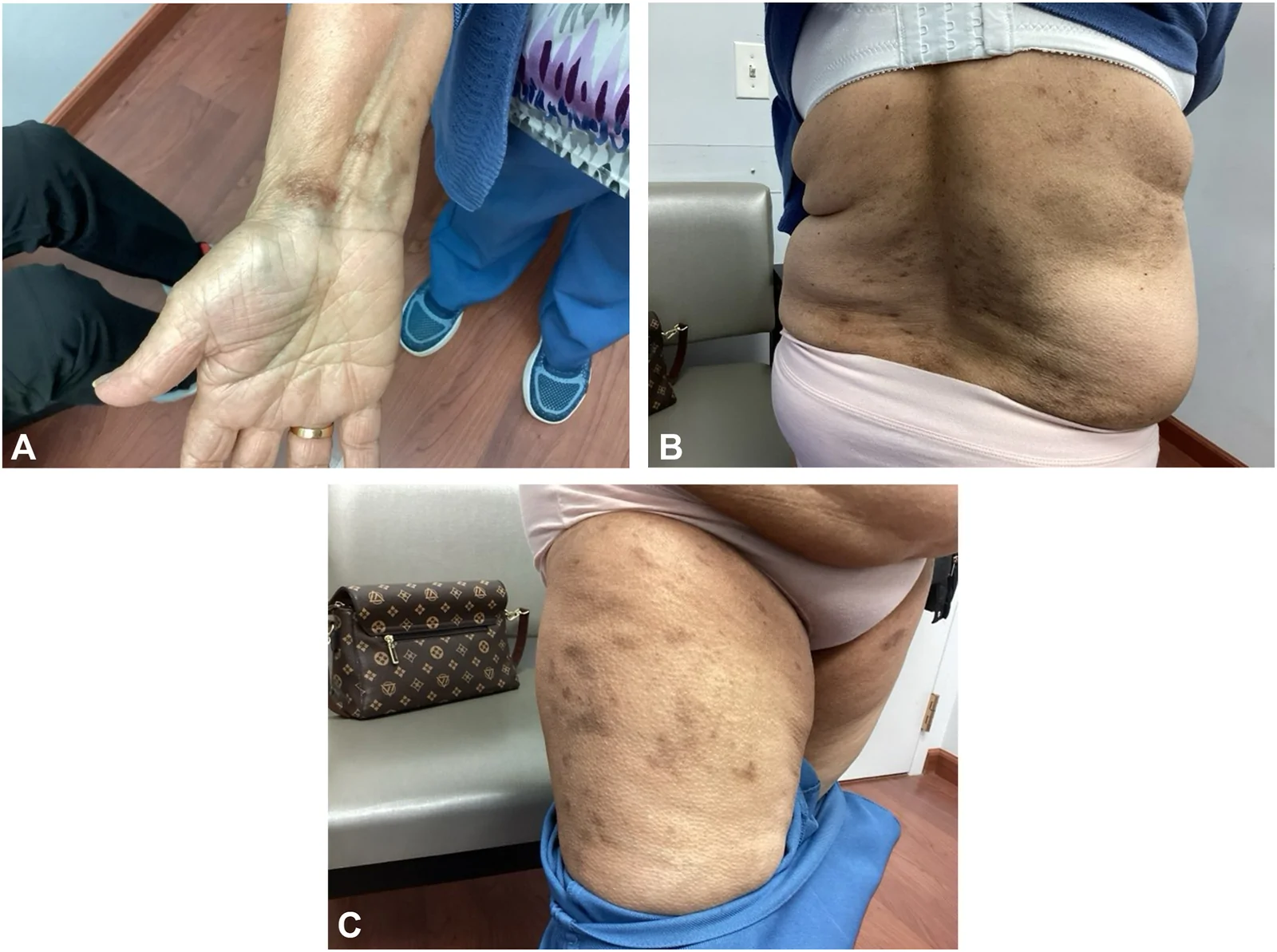
Improvement of lesions with deucravacitinib at 12-week follow-up. **A,** Lesions on the right ventrolateral wrist. **B,** Lesions diffusely on the back. **C,** Lesions on the bilateral thighs.

## Discussion

LP occurs more commonly in women, with a higher incidence in people of South Asian descent and has been linked to hepatitis C seropositivity.^1^ Lesions are frequently observed on the wrists, lower back, and ankles.^1^ If LP does not resolve within 1 to 2 years, recurrence may occur, leading to persistent PIH, especially in SoC.^1^ Although the usual presentation of LP is violaceous, SoC LP lesions can present as slate-gray or brown.^6^^,^^7^

The first-line treatment for cutaneous LP is topical class I corticosteroids applied twice daily for 2–5 weeks, with intralesional steroid injections reserved for refractory lesions.^1^ If diffuse, a daily prednisone taper can be given for 2 to 6 weeks.^1^ Second-line therapies include antibiotics (eg, metronidazole or sulfasalazine), isotretinoin, light therapy, and immunosuppressive agents (eg, methotrexate, etanercept, or adalimumab).^1^ Refractory LP may cause persistent pruritus and disfiguring PIH, particularly in SoC. In geriatric patients with diffuse disease, extensive topical therapy may be burdensome. We therefore selected deucravacitinib to reduce pruritus and PIH as a more feasible alternative to standard therapies.

While the pathogenesis of LP remains unclear, it is believed to rsepresent a T-cell mediated autoimmune disease involving IL-23 and Th17, resulting in keratinocyte apoptosis and necrosis.^1^^,^^2^ Deucravacitinib selectively inhibits TYK2, attenuating IL-23 signaling, decreasing Th17 proliferation, and downregulating inflammatory mediators like TNF-α and IL-17.^8^ This increased target specificity may contribute to its more favorable safety profile compared with other JAK inhibitors.^2^^,^^3^

Emerging literature highlights deucravacitinib as a promising treatment for LP. Ball et al outlined a case of extensive LP (80% BSA) unresponsive to triamcinolone and hydroxyzine with significant improvement in BSA (20%), pruritus, and erythema after 2 months of deucravacitinib.^2^ Another patient with diffuse hypertrophic cutaneous LP experienced notable plaque regression after 1.5 months of deucravacitnib combined with clobetasol and salicylic acid.^3^ Stolte et al further described 3 patients with chronic, erosive oral LP (OLP), 2 of whom achieved complete resolution of mucosal lesions, while the third demonstrated a considerable reduction in erosive and reticular lesions after 12 weeks of deucravatinib therapy.^4^ Similarly, another patient with OLP showed significant improvement in erythema and ulcerations after 5 months of deucravacitinib.^5^

Beyond LP, randomized phase III psoriasis trials showed meaningful improvements in pruritus with deucravacitinib, with benefits observed as early as week 1 and itch showing the greatest improvement among Psoriasis Symptoms and Signs Diary domains.^9^ Likewise, rapid pruritus relief has been demonstrated in pityriasis rubra pilaris and cutaneous lupus erythematosus, often after failure of prior therapies, suggesting this benefit may extend across inflammatory dermatoses. These observations parallel our patient’s rapid symptomatic improvement and support deucravacitinib when prompt pruritus relief is the predominant treatment goal.^10^

Together, the available LP literature and reports of rapid pruritus relief in other inflammatory dermatoses suggest that deucravacitinib is a promising treatment for refractory LP. To our knowledge, only 6 PubMed-indexed cases of LP treated with deucravacitinib have been reported.^2^, ^3^, ^4^, ^5^ Across all 6 patients, treatment was successful without reported adverse events.^2^, ^3^, ^4^, ^5^ Notably, most previously reported cases do not include patients with SoC, underscoring the unique contribution of our case. Larger studies are warranted to further evaluate its efficacy and safety.

## Conflicts of interest

None disclosed.

## References

[1] Arnold D.L., Syed H.A., Krishnamurthy K. (2026).

[2] Ball G.D., Golant A. (2024). A case of extensive Lichen Planus treated with deucravacitinib. Cureus.

[3] Sood S., Yadav G. (2024). Treatment of hypertrophic lichen planus using deucravacitinib. JAAD Case Rep.

[4] Stolte K.N., Mesas-Fernández A., Meier K. (2024). TYK2 inhibition with deucravacitinib ameliorates erosive oral lichen planus. Exp Dermatol.

[5] Vu M., Abdin R., Issa N.T. (2024). Treatment of oral lichen planus using deucravacitinib. JAAD Case Rep.

[6] Chung I.Y., Wong C.C., Rodrigues M.A. (2021). The importance of dermoscopy in subclinical lichen planus in skin of color. Int J Women's Dermatol.

[7] Diop A., Ly F., Ndiaye M.T. (2020). Epidemiology, clinical features, and associated factors in 78 cases of lichen planus on black skin. Int J Dermatol.

[8] Kalola U.K., Alberty A., Sheehy C. (2026).

[9] Armstrong A.W., Augustin M., Beaumont J.L. (2024). Deucravacitinib improves patient-reported outcomes in patients with moderate to severe psoriasis: results from the phase 3 randomized POETYK PSO-1 and PSO-2 trials. Dermatol Ther (Heidelb).

[10] D'Agostino M., Potestio L., Tommasino N., Megna M. (2026). Deucravacitinib in dermatology: current indication and existing off-label data. Drugs Context.

